# Serial coherent diffraction imaging of dynamic samples based on inter-frame continuity

**DOI:** 10.1038/s41377-025-01860-8

**Published:** 2025-07-01

**Authors:** Pengju Sheng, Fucai Zhang

**Affiliations:** Department of Electronic and Electrical Engineering, Southern University of Science and Technology (SUSTech), Shenzhen, China

**Keywords:** Imaging and sensing, Microscopy

## Abstract

Coherent diffraction imaging (CDI) provides lens-free imaging with diffraction-limited resolution and has become an important imaging modality at synchrotron facilities worldwide. The performance of current CDI approaches remains limited, particularly in their ability to handle dynamic samples or achieve consistent high-quality reconstructions. Here, we propose a novel coherent imaging approach for dynamic samples, which exploits the inter-frame continuity of the sample’s local structures as an additional constraint in phasing a sequence of diffraction patterns. Our algorithm incorporates an adaptive similarity determination procedure, eliminating the requirement for invariant regions in the sample and ensuring broad applicability to diverse sample types. We demonstrated the feasibility of this technique through experiments on various dynamic samples, achieving high-fidelity reconstructions within a few hundred iterations. With the same simple setup as conventional CDI, high image quality, and the ability to separate the sample transmission from its illumination probe, our method has the potential to significantly advance X-ray imaging and electron microscopy techniques for dynamic sample analysis.

## Introduction

Imaging techniques and microscopy play a pivotal role in scientific and industrial applications. Short-wavelength radiations, such as X-rays and high-energy electrons, are the primary tools for achieving nanometer to atomic-scale imaging resolution. Despite steady advancements in high-quality optical components for short-wavelength radiations, significant challenges remain. Fresnel zone plates with sub-ten nanometer outermost zones, Laue mirrors for X-rays, and aberration correctors for high-energy electrons have improved performance but still fall short of the prevalent demands in materials science and biology. Further enhancement of these components is hampered by the lack of suitable materials and the current limitations of fabrication technology.

Over the past 30 years, rapid growth has been seen in lensless coherent diffraction imaging (CDI) techniques^1–5^. In the framework of traditional CDI, a far-field diffraction pattern of the sample is acquired, and phase retrieval algorithms are used to retrieve the phase information of the diffraction pattern. We refer to this kind of technique as single-shot CDI. As pointed out by Miao^1^, once the continuous diffraction pattern of a non-periodic sample is sampled more finely than its Nyquist rate, it is possible to reverse the diffraction pattern to obtain the image of the sample. The most used phase retrieval algorithms originated from the Gerchberg-Saxton and Fienup algorithms^6–8^. Those algorithms could perform well for certain types of samples and data, but they are likely to fail for general complex samples.

A key milestone of CDI is the development of ptychography^9–12^. Ptychography leverages the spatial overlap constraint in the object plane, which significantly improves the iterative phasing algorithm’s convergence and has significantly impacted many fields^13–17^. During the data acquisition process of ptychography, the overlap in the scan positions leads to considerable redundant information in the acquired diffraction patterns. This redundancy effectively mitigates the twin-image ambiguity encountered in phase retrieval algorithms. It also relaxes the requirements of experimental conditions, such as accurate knowledge of scanning positions^18–20^ and illumination coherence^21–23^.

Due to the inherent requirement of sample scanning in ptychography, its application in dynamic imaging is limited. There have been attempts to extend the application of ptychography to dynamic samples^24–26^, but these efforts have not yet met expectations. The emergence and advancements of coherent modulation imaging^27–30^ offer an alternative approach for high-quality single-shot CDI. The imaging quality and algorithmic convergence are significantly improved by placing a pre-calibrated modulator downstream of the sample. However, this method requires prior measurement of the modulator, increasing the complexity of the system.

Methods that exploit the temporal continuity of dynamic samples^31–37^ have introduced new possibilities in the field of CDI, enabling the study of time-resolved processes with better imaging quality and precision. Recently, several studies have proposed leveraging the consistency of the partial structure of the sample as a novel constraint for CDI^31–34^. Tao et al. ^31^ and Lo et al. ^34^ introduced approaches that partition the sample plane into stationary and dynamic regions. They achieved improved algorithmic convergence by employing the stationary region as a consistent constraint across sequential frame reconstructions. However, this strategy increases the complexity of the experiment, limits the field of view, and necessitates prior information about the stationary region. Hinsley et al. ^33^ proposed a method based on standard deviation and hard thresholding to identify stationary regions within the sample plane. Still, distinct boundaries of the stationary regions are required to facilitate effective discrimination in their simulation. Nayer et al. ^37^ advanced this concept into a more practical approach by utilizing low rank as a constraint between frames, thus introducing the concept of low-rank phase retrieval. Nonetheless, this method necessitates the encoding of exit waves with a binary mask and lacks experimental validation in CDI.

For a video recording of an object, a significant portion of the object exit waves would remain structurally similar during the acquisition if the acquisition is faster than the sample’s variation rate. We denoted this as inter-frame continuity in this paper. Like probe overlap in ptychography, temporal consistency of local structures within the sample across different measurements can enhance the data redundancy, thereby mitigating twin-image ambiguity.

Here, we propose a novel method called serialCDI, which can rapidly reconstruct a time-varying object from its serially recorded far-field diffraction patterns. SerialCDI exploits the inter-frame continuity constraint using an adaptive similarity matrix $S({\boldsymbol{r}})$, where ${\boldsymbol{r}}$ denotes the coordinate vector in the sample plane, eliminating the need for pre-measured stationary regions or clearly defined boundaries. For the first time, we validated the robustness of the inter-frame continuity constraint against data loss and complex samples through experiments and simulations. Additionally, we demonstrated the ability to separate illumination from the sample distribution, enabling dynamic and quantitative measurement of the sample’s complex transmittance.

## Results

### The inter-frame continuity constraint

The traditional CDI phase retrieval algorithms iteratively update the object wave by alternating between the sample and detector planes, applying support and modulus constraints at each step. Previous approaches^31–34^ introduce a new constraint by leveraging the consistency of the sample’s local structure. Specifically, a binary mask $D({\boldsymbol{r}})$, with values of 0 and 1, partitions the sample plane into time-varying and stationary regions, respectively. During the reconstruction, points in the sample plane where $D\left({\boldsymbol{r}}\right)=1$ are replaced by their temporal averages. However, when sample variations are minimal, or boundaries are ambiguous, direct averaging can obscure subtle changes, leading to incorrect boundaries and stagnation in the phase retrieval algorithm.

SerialCDI advances the conventional method of partitioning the sample into time-varying and stationary regions by integrating the inter-frame continuity constraint through weighted average, offering a more general and robust solution. The method employs an adaptive similarity matrix $S\left({\boldsymbol{r}}\right)$, with values ranging continuously from 0 to 1. Values in $S({\boldsymbol{r}})$ approach 1 for points with minimal changes in their updated estimates across time instants, indicating that these points can be effectively replaced by their temporal averages. Conversely, points with significant changes are assigned values closer to 0. This continuous weighting significantly enhances the effectiveness of the inter-frame continuity constraint.

Standard deviation is used to quantify the degree of variation, with higher standard deviation indicating significant changes over time and lower standard deviation suggesting minimal changes. However, in the early stage of phase retrieval, before convergence, each frame’s reconstruction will be subject to strong artifacts, causing all points of the object estimate to exhibit significant standard deviation. To address this issue, the matrix $S({\boldsymbol{r}})$ must be updated adaptively as iteration progresses. SerialCDI has found an effective method to update $S\left({\boldsymbol{r}}\right)$, allowing the algorithm to fully leverage inter-frame continuity constraint to accelerate convergence without requiring any prior knowledge of the sample. Accordingly, the inter-frame continuity constraint can be enacted as:

1 $${\psi }_{j}\text{'}\left({\boldsymbol{r}};{t}_{n}\right)={S}_{j}\left({\boldsymbol{r}}\right)\overline{{\psi }_{j}}\left({\boldsymbol{r}}\right)+[1-{S}_{j}({\boldsymbol{r}})]{\psi }_{j}\left({\boldsymbol{r}};{t}_{n}\right)$$

where $n=1,2,\ldots ,N$; $N$ is the total number of frames; $j$ is the iteration number; ${\psi }_{j}\text{'}\left({\boldsymbol{r}};{t}_{n}\right)$ is the updated complex wave distribution at the $j$ th iteration; ${t}_{n}$ is the time instant; $\overline{{\psi }_{j}}\left({\boldsymbol{r}}\right)$ is the temporal average of ${\psi }_{j}\left({\boldsymbol{r}};{t}_{n}\right)$ and ${S}_{j}\left({\boldsymbol{r}}\right)$ is the similarity matrix calculated at the $j$ th iteration.

In the serialCDI algorithmic flow, after applying the support constraint, the inter-frame continuity constraint is imposed, followed by the modulus constraint. Additionally, the difference map update formula^5^ is adopted in the serialCDI algorithm. The strategy employed in serialCDI to update ${S}_{j}\left({\boldsymbol{r}}\right)$ will be detailed in the “Materials and methods” section.

### Imaging the growth of a thick crystal sample

**Fig. 1:**
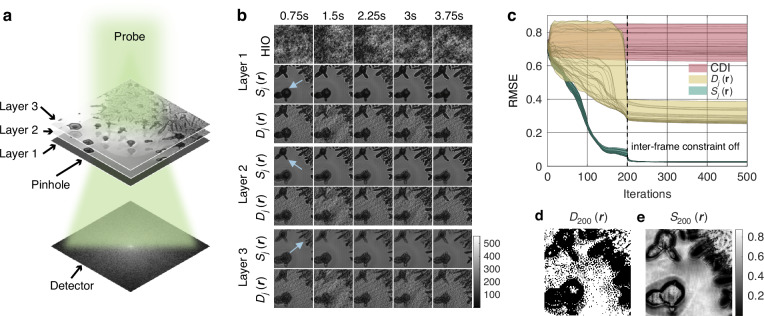
**Simulation of serialCDI and HIO of the thick crystal growth process**. **a** Schematic of the simulation setup. The incident probe sequentially passes through Layer 3, Layer 2, and Layer 1. **b** Reconstruction results of three layers, from the top to the bottom displays the reconstruction results of HIO, serialCDI with ${S}_{j}({\boldsymbol{r}})$, and serialCDI with ${D}_{j}({\boldsymbol{r}})$ with a time interval of 0.75 s, respectively. Blue arrows indicated the focal spots. **c** The RMSE of all exit waves. **d**, **e** The coefficient matrices ${D}_{200}({\boldsymbol{r}})$ and ${S}_{200}\left({\boldsymbol{r}}\right)$, respectively

To validate the reliability of serialCDI in reconstructing dynamic samples, we conducted a numerical simulation of imaging the growth process of a crystal sample. The sample consists of three layers with a 3 ${mm}$ separation, as shown in Fig. 1a. A set of 30 far-field diffraction patterns, each with dimensions of 512 × 512 pixels, was simulated using a wavelength of 518.6 ${nm}$, a detector pixel size of 6.5 $\mu m$ and a frame rate of 8 fps (frames per second). Poisson noise was added to the diffraction patterns. The planar illumination was formed by a 3 ${mm}$ diameter pinhole, with an average flux of $1.5\times {10}^{9}$ photons. In this simulation, only the exit waves at the pinhole plane were reconstructed for comparison. The invariant normalized error metric (RMSE) proposed by Fienup^38^. was used to monitor convergence.

To illustrate the significance of employing a continuous ${S}_{j}({\boldsymbol{r}})$, we compared the reconstructions obtained from Hybrid Input-Output^8^ (HIO, $\beta =0.5$), serialCDI with continuous ${S}_{j}({\boldsymbol{r}})$ and a binary mask ${D}_{j}\left({\boldsymbol{r}}\right)$. The serialCDI algorithm was run for a total of 500 iterations, with the inter-frame continuity constraint and the difference map updating formula disabled at the 200th iteration. The enlarged reconstruction results are shown in Fig. 1b. Layer2 and Layer3 are generated by back-propagating Layer1 by 3 ${mm}$ and 6 ${mm}$, respectively. As indicated by the blue arrows, Layer1 is focused in the lower-left corner, Layer2 is in the upper-left corner, and Layer3 is in the upper-right corner.

The convergence curve in Fig. 1c and the reconstruction results in Fig. 1b show that serialCDI has significantly improved convergence over HIO, with RMSE dropping below 0.02 at the 300th iteration, while HIO stagnated completely, showing strong artifacts.

The binary mask ${D}_{j}\left({\boldsymbol{r}}\right)$ assigns values of 0 and 1, segmenting the sample plane into variant and invariant regions, as shown in Fig. 1d. As indicated by Fig. 1e, ${S}_{j}({\boldsymbol{r}})$ accurately captures the variations within the samples, while ${D}_{j}\left({\boldsymbol{r}}\right)$ leads to incorrect boundaries. The convergence curve in Fig. 1c further demonstrates that employing ${S}_{j}({\boldsymbol{r}})$ during the reconstruction process significantly accelerates the algorithm. This acceleration is attributed to the substantial artifacts in the early stages of reconstruction, which cause slowly varying regions to exhibit large standard deviations. A continuous ${S}_{j}({\boldsymbol{r}})$, being less sensitive to noise, is crucial at this stage to mitigate the effects of noise.

Furthermore, the binary ${D}_{j}\left({\boldsymbol{r}}\right)$ leads to points with subtle changes being directly averaged, resulting in erroneous results. Employing a continuously varying ${S}_{j}({\boldsymbol{r}})$ avoids direct averaging, thereby preventing such inaccuracies. As shown in the serialCDI reconstruction results of Layer1 in Fig. 1b, diffraction rings caused by forward propagation of Layer3 are evident in the upper-right corner. In contrast, these rings disappear in the Layer1 obtained by the ${D}_{j}\left({\boldsymbol{r}}\right)$, leading to a loss of fine structural details. As a result, after the back propagation of Layer1, the reconstruction results of Layer2 and Layer3 obtained by the ${D}_{j}\left({\boldsymbol{r}}\right)$ become blurred. In contrast, serialCDI preserves and accurately retrieves these structures, demonstrating its advantage in resolving dynamic sample features.

### Observation of the swimming behavior of euglenids

To demonstrate the principle, we designed three experiments to showcase the imaging capability of serialCDI for dynamic samples. In the first experiment, a planar wave with a wavelength of 518.6 ${nm}$ was used to illuminate swimming euglenids with a typical size ranging from 10 $\mu m$ to 40 $\mu m$. A 10$\times$ magnification optic was placed downstream of the sample to facilitate the observation of fine structures of the euglenids. After magnification, the transmitted wave through the sample was directed onto a pinhole. This wave then passed through a Fourier lens with a focal length of 75 ${mm}$, and a 14-bit camera positioned at its back focal plane captured diffraction patterns with 512 × 512 pixels, with a pixel size of 6.5 $\mu m$, at a rate of 100 fps and an exposure time of 5.43 ${ms}$. The exposure time was pre-calculated to ensure that the translation of the euglenids remained within one pixel throughout the exposure, avoiding motion blur. A total of 150 diffraction patterns of the sample and one diffraction pattern of the probe were captured.

**Fig. 2:**
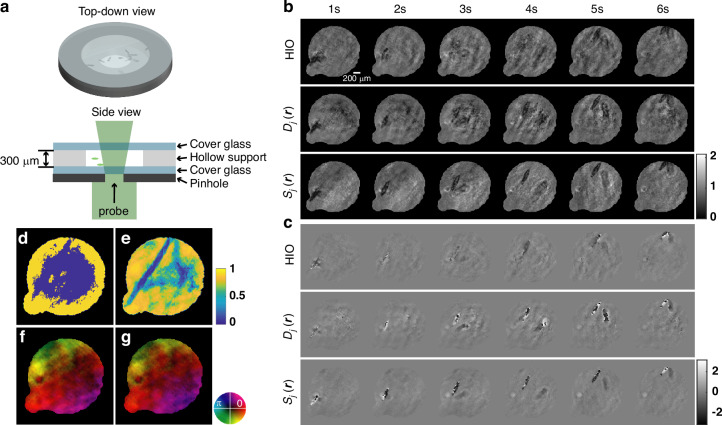
**Experimental results of the serialCDI with a biological sample**. **a** Diagram of the sample container. **b** The amplitude distributions reconstructed by HIO (first row), serialCDI with binary mask ${D}_{j}\left({\boldsymbol{r}}\right)$ (second row) and serialCDI with a continuous similarity ${S}_{j}\left({\boldsymbol{r}}\right)$ (third row) with a time interval of 1 s. **c** The corresponding phase of (**b**). **d**, **e** Binary mask ${D}_{200}\left({\boldsymbol{r}}\right)$ and degree of similarity ${S}_{200}\left({\boldsymbol{r}}\right)$ at the 200th iteration, the black region represents the support area. **f**, **g** Probe reconstructed by serialCDI with a binary mask ${D}_{j}\left({\boldsymbol{r}}\right)$ and serialCDI with a continuous similarity ${S}_{j}\left({\boldsymbol{r}}\right)$ respectively. Scale bar: 200$\mu m$

Figure 2a illustrates the structure of the sample container, which consists of two cover glasses and a hollow support. The hollow support has an approximate thickness of 300 $\mu m$. Figures 2b, c show the reconstruction results after 250 iterations under three conditions: HIO (${\rm{\beta }}=0.5$), serialCDI with a binary mask ${D}_{j}({\boldsymbol{r}})$, and serialCDI with a continuous similarity ${S}_{j}\left({\boldsymbol{r}}\right)$. The inter-frame continuity constraint is disabled at the 200th iteration. For more reconstruction details, please refer to Supplementary Movie S1.

From the serialCDI results, it is evident that two euglenids are captured within the field of view. In contrast, in the HIO reconstruction, noise in the background makes the second euglenid nearly invisible. Similarly, in the serialCDI with a binary mask, the reconstruction quality is only slightly better than that of the HIO. This is because the algorithm fails to accurately distinguish the invariant regions during iterations, as shown in Fig. 2d.

As shown in Fig. 2e, the similarity ${S}_{200}\left({\boldsymbol{r}}\right)$ obtained by serialCDI has lower values along the motion trajectory of the second euglenid compared to other areas. This demonstrates that serialCDI effectively captures dynamic changes in the sample, enabling improved identification of the second euglenid. A similar phenomenon can also be observed in the reconstructed phase results shown in Fig. 2c. Due to the simple structure of the plane wave, the reconstruction quality of probes is similar for both methods, as shown in Fig. 2f, g. The global phase gradient in the reconstructed probe could be caused by the tilted illumination or the shifts in diffraction patterns.

**Fig. 3:**
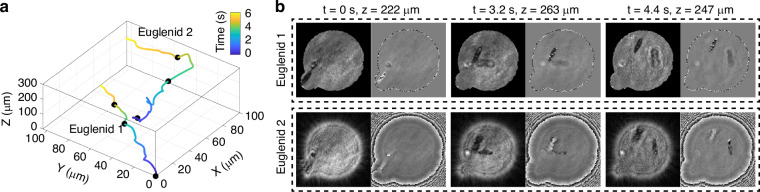
**Motion trajectories in three-dimensional space and refocused images of the euglenid**. **a** Motion trajectory of euglenids in three-dimensional space with color bar represents time. **b** Refocused images of euglenids. The value *z* at the top indicates the propagation distance along the direction of light

By performing simulated propagation on the reconstruction results, we can obtain clear images of the second euglenid and plot their motion trajectories in three-dimensional space, as shown in Fig. 3. Figure 3a illustrates the 3D motion trajectories of two euglenids, with coordinates measured in micrometers and the color bar representing time. The positive Z-axis was defined as the direction of light propagation in the experiment. From Supplementary Movie S1, it is evident that the motion trajectory of the euglenids is helical. Figure 3b shows the euglenid images of the three black markers indicated in Fig. 3a.

**Fig. 4:**
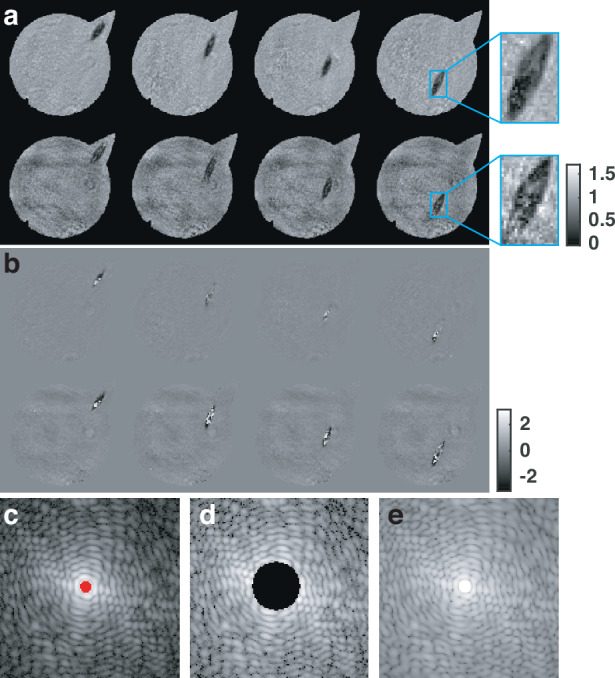
**Robustness of serialCDI to data missing.** The diffraction patterns are shown in the log scale. **a** Reconstructed amplitudes from data **c** and data **d**, respectively. **b** Corresponding reconstructed phases. **c** Camera-captured diffraction pattern and red pixels indicate overexposed pixels. **d** Artificially set missing pixels, ~910 pixels (25 speckles). **e** Simulated diffraction pattern using reconstructed result

Given the redundant information in the continuously recorded diffraction patterns, serialCDI is highly robust to data loss. To demonstrate this, we conducted another experiment using euglenid. In the second experiment, 150 diffraction patterns with a size of 512 × 512 pixels were captured. Due to the weak scattering of light by the euglenids, most of the light was concentrated in the center. To enhance the signal-to-noise ratio of high-frequency information, the central region of the diffraction pattern was intentionally overexposed, covering ~50 pixels, as indicated by the red pixels in Fig. 4c. Then we artificially removed ~910 pixels (25 speckles) from the center of the diffraction pattern to simulate the missing data situation and used this modified pattern (Fig. 4d) for reconstruction. The reconstructed results after 1500 iterations are shown in Fig. 4a. The top row of Fig. 4a shows the reconstruction results with no pixel loss (Fig. 4c), while the second row shows the results reconstructed from the diffraction pattern with missing data (Fig. 4d).

Figure 4a, b and Supplementary Movie S2 show that even with the loss of nearly 910 pixels, the serialCDI algorithm successfully reconstructed the sample’s amplitude and phase. Although the quality of the reconstructed amplitude declines slightly, it remains acceptable. Additionally, the missing central information does not significantly impact the phase reconstruction.

Figure 4e displays the simulated diffraction pattern generated from the reconstructed sample and illumination probe. It shows that the missing pixels in the diffraction pattern center have been well-recovered, with minimal difference from the original diffraction pattern (Fig. 4c). This further confirms the robustness of the continuously recorded diffraction patterns to data loss.

### Potential application on X-ray free electron laser

**Fig. 5:**
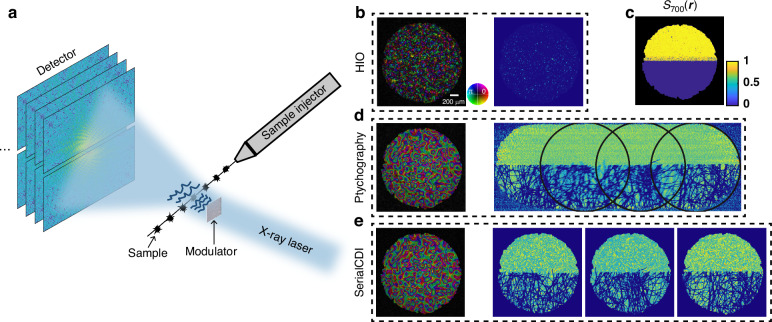
**Visible light experiment simulating potential X-ray experiment with liquid jet sample delivery system**. **a** Schematic of XFEL experiment with a liquid jet sample delivery system. **b,** **d** and **e** Reconstructed objects and probes of a laterally translated sample using HIO, ptychography, and serialCDI. **c** Similarity matrix ${S}_{700}\left({\boldsymbol{r}}\right)$, the black region represents the support area. Scale bar: 200 μm

When the rate of change of the sample considerably exceeds camera acquisition speed, resulting in weak continuity between consecutive frames, the inter-frame continuity constraint can still remain effective. This is achieved by introducing structured illumination in the object plane, which encodes additional spatial information to link successive frames. As shown in Fig. 5a, in the application scenario of X-ray free electron laser (XFEL), the sample is ejected by an injector, and multiple diffraction patterns are captured in rapid succession. Because the samples differ between shots and are typically smaller than the beam diameter, serialCDI can effectively utilize the structured illumination to reconstruct these dynamic samples with high fidelity.

To validate that serialCDI remains effective in scenarios with weak inter-frame continuity, such as in XFEL applications, we conducted a visible-light validation experiment using an optical setup analogous to the setup in Fig. 5a. In the experiment, a tissue was laterally moved to simulate a dynamic sample, and the camera captured diffraction patterns at a frame rate of 1 fps. The tissue’s lateral movement caused significant variations at the same point on the sample plane, resembling the challenges in XFEL applications. The sample was illuminated using structured illumination with a wavelength of 520 ${nm}$ and a diameter of 2.0 ${mm}$, and the structured illumination was generated by a randomly distributed modulator placed in front of the pinhole. Downstream of the sample was a lens with a focal length of 75 ${mm}$ to simulate the far-field recording condition. At the back focal plane of the lens was a 14-bit detector with a pixel size of 6.5 $\mu m$, on which 60 diffraction patterns of the sample and a diffraction pattern of the probe, all with 800 × 800 pixels, were collected.

Figure 5b, d and e presents the reconstructed objects and probes using HIO, ptychography and serialCDI after 2000 iterations, and the inter-frame continuity constraint is disabled at the 700th iteration. In comparison, Fig. 5b demonstrates that HIO fails to converge for such complex samples. SerialCDI provides significantly improved image quality compared to HIO, both in terms of the reconstruction results of objects and probe.

As shown in Fig. 5c, the lower half of ${S}_{700}({\boldsymbol{r}})$, which is occupied by the moving tissue, exhibits a low similarity coefficient, indicating that there is no similarity between the different locations of tissue. This suggests that the lateral movement of the tissue effectively simulates the scenario where there is no continuity between consecutive samples, as encountered in XFEL applications.

To ensure convergence in the ptychography reconstruction, a support constraint and a modulus constraint were applied to the probe using the same support as the object and the diffraction pattern of the probe. Figure 5e shows the reconstruction results of serialCDI indicated by the black circles in Fig. 5d, corresponding to the time instances 20 s, 40 s, and 60 s, respectively. SerialCDI, without relying on overlapping scanning positions, enables imaging of moving samples or time-resolved processes while achieving reconstruction quality comparable to ptychography. As shown in Fig. 5d, e, the reconstruction quality of the probes in both methods is similar. Ptychography exhibits dynamic blur in the middle of the reconstructed sample due to instabilities during the scanning process. In contrast, serialCDI, a dynamic imaging technique, does not exhibit such blur. This further demonstrates the advantage of serialCDI for dynamic imaging. The noisier background in the reconstructed objects by serialCDI is due to the lack of averaging effect, as introduced in ptychography through scanning.

The introduction of uncharacterized structured illumination increased the complexity of the exit waves; however, it effectively accelerates algorithm convergence by enforcing a strong inter-frame continuity constraint and enhancing the signal-to-noise ratio of the diffraction patterns. As shown in Supplementary Movie S3, the energy of the diffraction pattern spread out from the center, resulting in an increased signal-to-noise ratio in the high-frequency regions.

The ability of serialCDI to observe dynamic samples is only limited by the acquisition speed of the camera, while dynamic blur can be eliminated by short exposure time or short pulse duration. As a result, serialCDI is well-suited for XFEL, taking advantage of its short pulse duration and high brightness.

## Discussion

The inter-frame continuity constraint is similar to the overlap constraint in ptychography. The difference is that the inter-frame continuity is a natural constraint available in many cases, in contrast to ptychography, whose overlap constraint is introduced through scanning, limiting its application for dynamic samples. SerialCDI achieves dynamic imaging of samples by utilizing the continuity between frames to construct overlapping, thereby avoiding scanning.

**Fig. 6:**
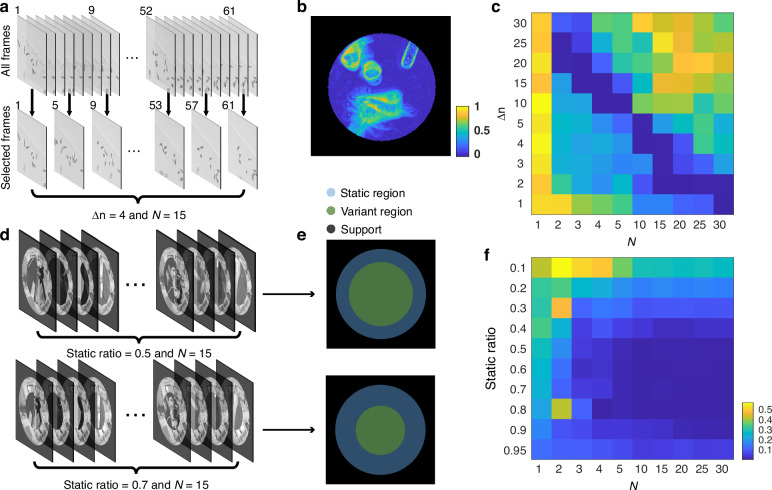
**Stress tests of serial CID**. **a** Illustration of the data generation process for stress test 1. **b** Normalized standard deviation for case with ∆*n* = 4 and *N* = 15. **c** RMSE distribution of stress test 1. **d** Illustration of the data generation process for stress test 2. **e** Simulated objects of stress test 2. **f** RMSE distribution of stress test 2

To evaluate the robustness of serialCDI against the inter-frame continuity, we conducted stress tests in two scenarios. In the first test, we downsampled an euglenid video with varying frame intervals ${{\Delta }}n$ and number of frames ($N$) to generate simulated diffraction patterns. Figure 6a presents an example with $\Delta n=4$ and $N=15$, where $\Delta n$ denotes the gap between selected frames, and $N$ represents the total number of frames selected for a given frame interval $\Delta n$. By varying the $\Delta n$, the inter-frame continuity between consecutive frames changes. For each condition, we conducted 20 independent reconstructions using random initial guesses. The average RMSE values are presented in Fig. 6c.

As shown in the RMSE distribution in Fig. 6c, as $N$ increases, a smaller $\Delta n$ is required to achieve a lower RMSE. When fewer frames are used, serialCDI can accommodate a broader range of $\Delta n$. In other words, with more diffraction patterns, the camera must capture frames more quickly to maintain inter-frame continuity. If the sample changes rapidly, fewer diffraction patterns are needed, but this can affect the stability of serialCDI’s convergence. With fewer than five frames, serialCDI may either converge or stagnate quickly.

Figure 6b presents the normalized standard deviation for a well-performing case with $N=15$ and $\Delta n=4$. In this case, 50–60% of the regions yield a small standard deviation, indicating that those regions remain relatively consistent across those frames. Other cases with low RMSE values also demonstrate a similar 50–60% consistency.

Additionally, we generated a sample with static (blue) and variant regions (green) for the quantitative test, as illustrated in Fig. 6e. The variant region is formed by assigning it unrelated sample. As demonstrated in Fig. 6d, e, by varying the size of the static region, we can quantitatively study the impact of inter-frame continuity on the algorithm’s convergence. The static ratio, calculated as the area of the static region divided by the total illumination area, is directly correlated with inter-frame continuity.

As shown by the RMSE distribution in Fig. 6f, serialCDI achieves its lowest RMSE when the static ratio is around 50–60%, consistent with the results from the first stress test. SerialCDI can still converge even when up to 85% of the sample undergoes significant changes. This further demonstrates that serialCDI can effectively exploit the inter-frame continuity to accelerate algorithm convergence.

For serialCDI experiments, it is recommended to optimize the camera’s acquisition speed and adjust the number of frames to ensure that at least 50% of the sample’s regions show minimal temporal variation, thereby improving reconstruction accuracy.

In the presented results, all frames share one similarity matrix ${S}_{j}({\boldsymbol{r}})$. In the case of data collection of a long sequence of frames, for which significant changes in the sample structure could occur, a running time window can be used to select a fixed number of frames^39^.

To conclude, we propose an algorithm for imaging dynamic samples based on an adaptive similarity matrix, called serialCDI, which exhibits rapid convergence and high robustness to missing data in reconstructing experimental data of real samples. We have also demonstrated that the rate of observing dynamic samples depends solely on the camera’s acquisition rate. Moreover, short exposure time can reduce dynamic blur induced by sample vibration.

SerialCDI holds high potential for further improvements. Currently, serialCDI requires the sample exit wave to have clear boundaries. The shrink-wrap algorithm can mitigate this requirement^40^, but other improvements are still needed to make serialCDI applicable to extended samples. By combining multi-wavelength light sources with serialCDI, the imaging quality of current multi-wavelength CDI^41,42^ could be improved, facilitating dynamic imaging with capability of chemical resolution. Like ptychography, the serialCDI dataset has a high degree of redundancy. Hence, there is potential for implementing serialCDI with broadband light sources by adopting mixed states^23^, monochromatization^2^, polyCDI^43^, or deconvolution approaches^29^, thereby enhancing its suitability for transmission electron microscopy or XFEL, where partial coherence has been a principal limiting factor affecting imaging resolution^43,44^.

The proposed serialCDI method has a simple setup and requires no imaging optic. Therefore, it is applicable across the spectrum of light and particle radiation. A condenser lens with an upstream aperture can form the confined probe, providing a large working distance around the sample. Thus, serialCDI would be highly suitable for in-situ observation of dynamic samples in their native environment.

## Materials and methods

**Fig. 7:**
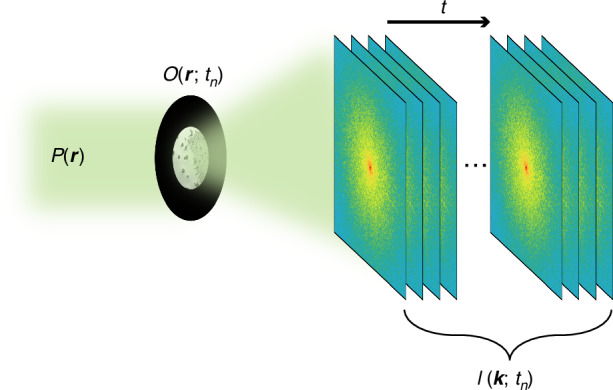
**Schematic layout of experimental setup**. The extent of dynamic sample $O({\boldsymbol{r}};{t}_{n})$ is confined by a pinhole; A sequence of far-field diffraction patterns, $I({\boldsymbol{k}};{t}_{n})$, are collected from a time-varying sample

Figure 7 is a schematic diagram of the experimental setup of SerialCDI, which is identical to the traditional CDI setup, so serialCDI could be conveniently implemented in broad scenarios. A confined probe $P({\boldsymbol{r}})$ is formed on the sample, i.e., by a pinhole. A time-varying object $O({\boldsymbol{r}};{t}_{n})$ is illuminated by $P({\boldsymbol{r}})$, where $n=1,2,\ldots ,N$; then the corresponding exit wave $\psi \left({\boldsymbol{r}};{t}_{n}\right)$, at various time instants ${t}_{n}$, propagate to the detector and their far-field diffraction patterns $I({\boldsymbol{k}};{t}_{n})$ are collected. Here ${\boldsymbol{k}}$ is the coordinate vector in the detector plane. In addition, a diffraction pattern of the probe, ${I}_{P}({\boldsymbol{k}})$, is also acquired.

**Fig. 8:**
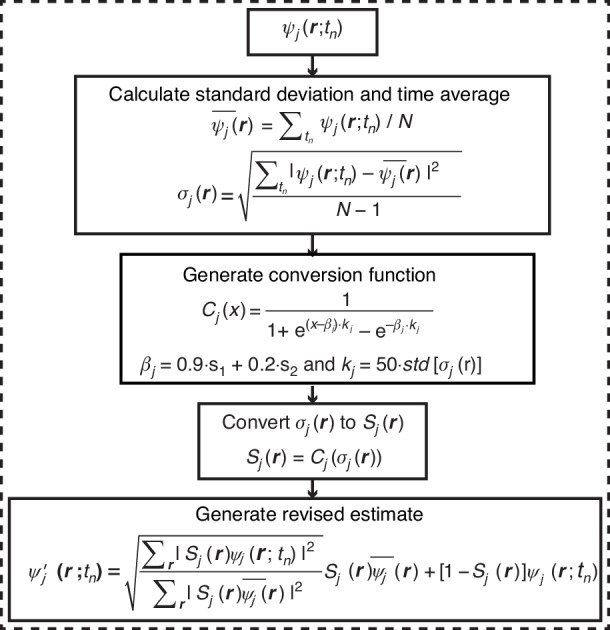
**Procedure for the inter-frame continuity constraint**. ${std}[* ]$ calculate the standard deviation of *

Figure 8 shows the procedure of the inter-frame continuity constraint. After applying the support constraint to all exit waves, the standard deviation map ${\sigma }_{j}\left({\boldsymbol{r}}\right)$ with respect to ${t}_{n}$ is utilized to measure the degree of variation between exit waves at each pixel.

2 $${\sigma }_{j}\left({\boldsymbol{r}}\right)=\sqrt{\frac{{\sum }_{{t}_{n}}{\left|{\psi }_{j}\left({\boldsymbol{r}};{t}_{n}\right)-{\bar{\psi }}_{j}\left({\boldsymbol{r}}\right)\right|}^{2}}{N-1}}$$

The standard deviation ${\sigma }_{j}\left({\boldsymbol{r}}\right)$ is then converted into ${S}_{j}({\boldsymbol{r}})$ by ${S}_{j}\left({\boldsymbol{r}}\right)={C}_{j}({\sigma }_{j}\left({\boldsymbol{r}}\right))$,

3 $${C}_{j}(x)=\frac{1}{1+{e}^{\left(x-{\beta }_{j}\right){\kappa }_{j}}-{e}^{-{\beta }_{j}{\kappa }_{j}}}$$

where $x$ is the independent variable. Equation (3) is derived from the primary form of $1/(1+{e}^{x})$, which has a flipped *S* shape. The coefficient is higher for points in the object plane with smaller standard deviations and vice versa. The term $-{e}^{-{\beta }_{j}{\kappa }_{j}}$ is used to ensure ${C}_{j}(x)$ reaches 1 when the standard deviation approaches zero. The values of ${\beta }_{j}$ and ${\kappa }_{j}$ are updated as the iteration progresses. To have a better robustness during iterations, ${\beta }_{j}$ is calculated as ${\beta }_{j}=0.9\cdot {s}_{1}+0.2\cdot {s}_{2}$ and ${\kappa }_{j}$ is set to 50 times the standard deviation of ${\sigma }_{j}\left({\boldsymbol{r}}\right)$. Specifically, the points in ${\sigma }_{j}\left({\boldsymbol{r}}\right)$ that satisfy the support constraint are extracted and collected into a column vector, which is denoted as ${\sigma }_{c}(x)$. Then ${\kappa }_{j}=50\cdot \sqrt{{\sum }_{x}{\left|{\sigma }_{c}\left(x\right)-\bar{{\sigma }_{c}}\right|}^{2}/({N}_{{\rm{s}}}-1)}$, where ${N}_{{\rm{s}}}$ is the number of elements in ${\sigma }_{c}$, and ${{\bar{\sigma}}_{c}}$ is the mean value of ${\sigma }_{c}\left(x\right)$.

In the reconstruction using a binary ${D}_{j}\left({\boldsymbol{r}}\right)$ shown in Fig.1 and Fig. 2, ${\kappa }_{j}$ is set to a large fixed value (>${10}^{5}$) to simulate hard thresholding. ${s}_{1}$ and ${s}_{2}$ are the averages of the lowest 10% and highest 10% elements of ${\sigma }_{j}\left({\boldsymbol{r}}\right)$. Using averages instead of the highest and lowest values can lead to a more stable convergence because the latter tends to vary wildly between iterations.

Then the revised estimate of exit waves can be generated by,

4 $${\psi }_{j}\text{'}\left({\boldsymbol{r}};{t}_{n}\right)=\sqrt{\frac{\sum _{{\boldsymbol{r}}}{\left|{S}_{j}\left({\boldsymbol{r}}\right){\psi }_{j}\left({\boldsymbol{r}};{t}_{n}\right)\right|}^{2}}{\sum _{{\boldsymbol{r}}}{\left|{S}_{j}\left({\boldsymbol{r}}\right){\bar{\psi }}_{j}\left({\boldsymbol{r}}\right)\right|}^{2}}}{S}_{j}\left({\boldsymbol{r}}\right){\bar{\psi }}_{j}\left({\boldsymbol{r}}\right)+[1-{S}_{j}\left({\boldsymbol{r}}\right)]{\psi }_{j}\left({\boldsymbol{r}};{t}_{n}\right)$$

Here, a factor is imposed on the term ${S}_{j}\left({\boldsymbol{r}}\right){\bar{\psi}}_{j}\left({\boldsymbol{r}}\right)$ to ensure energy conservation. After the inter-frame continuity constraint, we impose the modulus constraint to the revised exit wave guesses by taking the Fourier transform of it; replacing modulus and taking the inverse Fourier transform. The resulting exit wave guesses are used for the next iteration, and the iteration continues. After repeating these steps hundreds of times, the iteration usually reaches preliminary convergence. At this stage, initial guesses for both the objects and probe are generated. The initial guess for the probe is the time-average of ${\psi }_{j}\left({\boldsymbol{r}};{t}_{n}\right)$,

5 $${P}_{j}\left({\boldsymbol{r}}\right)={\bar{\psi }}_{j}\left({\boldsymbol{r}}\right)$$

The initial guess for the object is:

6 $${O}_{j}\left({\boldsymbol{r}};{t}_{n}\right)=\frac{{\psi }_{j}\left({\boldsymbol{r}};{t}_{n}\right)}{{P}_{j}\left({\boldsymbol{r}}\right)+\epsilon }$$

where $\epsilon$ is a small constant preventing division by zero. After generating the initial guesses for ${O}_{j}\left({\boldsymbol{r}};{t}_{n}\right)$ and ${P}_{j}\left({\boldsymbol{r}}\right)$, the following updates use the ePIE engine^11^ to separate the ${O}_{j}\left({\boldsymbol{r}};{t}_{n}\right)$ and ${P}_{j}\left({\boldsymbol{r}}\right)$ from the exit wave guesses,

7 $${O}_{j+1}({\boldsymbol{r}};\,{t}_{n})={O}_{j}({\boldsymbol{r}};\,{t}_{n})+{\alpha }_{1}\frac{{{P}_{j}}^{\ast }({\boldsymbol{r}})}{{|{{P}_{j}}^{\ast }({\boldsymbol{r}})|}_{max}^{2}}\cdot \Delta {\psi }_{j}$$

8 $${P}_{j+1}({\boldsymbol{r}})={P}_{j}({\boldsymbol{r}})+{\alpha }_{2}\frac{{{O}_{j}}^{\ast }({\boldsymbol{r}};\,{t}_{n})}{{|{{O}_{j}}^{\ast }({\boldsymbol{r}};\,{t}_{n})|}_{max}^{2}}\cdot \Delta {\psi }_{j}$$

where $\Delta {\psi }_{j}=[{\psi }_{j}{\rm{^{\prime} }}{\rm{^{\prime} }}({\boldsymbol{r}};{t}_{n})-{\psi }_{j}({\boldsymbol{r}};{t}_{n})]$, ${\psi }_{j}{\rm{^{\prime} }}{\rm{^{\prime} }}({\boldsymbol{r}};{t}_{n})$ is the exit wave guess after applying modulus constraint and ${\psi }_{j}\left({\boldsymbol{r}};{t}_{n}\right)$ is the exit wave guess before applying inter-frame constraint. The constants ${\alpha }_{1}$ and ${\alpha }_{2}$ alter the rate of updating. In this study both ${\alpha }_{1}$ and ${\alpha }_{2}$ are set to 0.5. In addition to being updated through the ePIE engine, ${P}_{j}\left({\boldsymbol{r}}\right)$ is also confined to the support constraint and the modulus constraint. Then, multiply the ${P}_{j+1}\left({\boldsymbol{r}}\right)$ and ${O}_{j+1}\left({\boldsymbol{r}};{t}_{n}\right)$ together to obtain ${\psi }_{j+1}\left({\boldsymbol{r}};{t}_{n}\right)$ for the next iteration and repeat the above steps until a predefined stop criterion is met. Here, we adopt the difference map^5^ updating formula in serialCDI ($\beta =-1$). The error reduction^7^ algorithm is utilized at the final stage of reconstruction to seek the global minimum.

## Supplementary information

Supplementary Information

Supplementary Movie S1

Supplementary Movie S2

Supplementary Movie S3

## Data Availability

Simulation and experimental data are available from the corresponding author upon reasonable request.
