# Supplementary material for: Serial coherent diffraction imaging of dynamic samples based on inter-frame continuity
Source: Light Sci Appl. 2025 Jul 1;14:230. doi: 10.1038/s41377-025-01860-8 (PMC12215372; doi:10.1038/s41377-025-01860-8)
Supplement: Supplementary file 1 — Supplementary Information [file 41377_2025_1860_MOESM1_ESM.docx]

Supplementary Information for

**Serial coherent diffraction imaging of dynamic samples based on inter-frame continuity**

**Pengju Sheng^1^, Fucai Zhang^1,*^**

*^1^Department of Electrical and Electronic Engineering, Southern University of Science and Technology (SUSTech), Shenzhen 518055, China*

[*^*^zhangfc@sustech.edu.cn*](mailto:*zhangfc@sustech.edu.cn)

This PDF file includes:

Movie S1. Experimental results of the serialCDI with biological samples.

Movie S2. Reconstruction results of serialCDI with data missing diffraction patterns.

Movie S3. Visible light experiment simulating potential x-ray experiment with liquid jet sample delivery system.
